# Real-life experiences with CAR T-cell therapy with idecabtagene vicleucel (ide-cel) for triple-class exposed relapsed/refractory multiple myeloma patients

**DOI:** 10.1186/s12885-023-10824-3

**Published:** 2023-04-15

**Authors:** Dilara Akhoundova Sanoyan, Katja Seipel, Ulrike Bacher, Marie-Noelle Kronig, Naomi Porret, Gertrud Wiedemann, Michael Daskalakis, Thomas Pabst

**Affiliations:** 1 Department of Medical Oncology, Inselspital, University Hospital of Bern, Center for Hemato-Oncology; University Cancer Center, Bern, 3010 Switzerland; 2 Department for Biomedical Research, University of Bern, Bern, 3008 Switzerland; 3 Department of Hematology and Central Hematology Laboratory, Inselspital, Bern University Hospital, University of Bern, Bern, Switzerland; 4 Clinical Genomics Lab, Inselspital, University Hospital of Bern, Bern, 3010 Switzerland

**Keywords:** Myeloma, CAR-T, Relapsed, Outcome, Ide-cel

## Abstract

**Background:**

Chimeric antigen receptor (CAR) T-cell therapy has revolutionized the treatment landscape of relapsed/refractory multiple myeloma (RRMM), leading to unprecedented responses in this patient population. Idecabtagene vicleucel (ide-cel) has been recently approved for treatment of triple-class exposed RRMM. We report real-life experiences with the commercial use of ide-cel in RRMM patients.

**Methods:**

We performed a retrospective analysis of the first 16 triple-class exposed RRMM patients treated with ide-cel at a single academic center. We assessed toxicities, response to treatment, CAR T expansion and soluble BCMA (sBCMA) levels.

**Results:**

We identified 16 consecutive RRMM patients treated with ide-cel between 06–10/2022. Median age was 69 years, 6 (38%) patients had high-risk cytogenetics, 3 (19%) R-ISS stage III, and 5 (31%) extramedullary disease. Median number of previous treatment lines was 6 (3–12). Manufacturing success rate was 88% (6% required second lymphapheresis, 6% received an out-of-specification product). At 3 months, the overall response rate (ORR) was 69% (44% sCR, 6% CR, 19% VGPR). Cytokine release syndrome (CRS) occurred in 15 (94%) patients (88% G1, 6% G2), immune effector-cell associated neurotoxicity syndrome (ICANS) in 1 (6% G1), febrile neutropenia in 11 (69%), and infections in 5 (31%). Prolonged hematologic toxicity occurred in 4/16 (25%) patients. Other non-hematological toxicities were elevated hepatic enzymes (38%), colitis (6%, G3) and DIC (6%, G2). Responses were more frequent in patients with higher CAR T expansion (100% vs 38%), and lack of decrease or plateau of sBCMA levels was typically observed in non-responders.

**Conclusions:**

We report one of the first cohorts of RRMM treated with commercial ide-cel. The ORR was 69% and safety profile was manageable, but prolonged hematologic toxicity still represents a major challenge. Responses correlated with in vivo CAR T cell expansion, underlining the need of further research to optimize CAR T expansion.

## Background

Chimeric antigen receptor (CAR) T-cell therapy is a novel cellular immunotherapy approach based on ex vivo genetic engineering of autologous or allogenic T cells, providing them with a new “artificial” surface receptor able to efficiently target a specific tumor antigen [1, 2]. The recognition and binding of the CAR to the tumor surface target leads to a potent immune activation, which is major histocompatibility complex -independent [1, 3]. Over the past years, CAR T-cell therapies have experienced a relevant development in the field of hematological malignancies [4–7]. Currently, four commercial CD19-targeting CAR T cell products are FDA approved for B-cell lymphoma and B-cell acute lymphoblastic leukemia: tisagenlecleucel (Kymriah®), axicabtagen ciloleucel (Yescarta®), brexucabtagen autoleucel (Tecartus®) and lisocabtagen maraleucel (Breyanzi®) [4, 6]. For multiple myeloma (MM), the majority of currently available CAR T-cell products target the B-cell maturation antigen (BCMA), also termed TNFRSF17, which is selectively expressed on mature B lymphocytes and has a relevant role for their survival and proliferation [8]. Two BCMA-targeting CAR T products—idecabtagene vicleucel (ide-cel, Abecma®) and, more recently, ciltacatagene-autocel (cilta-cel, Carvykti®) – have been to date FDA-approved [4, 9, 10]. Ide-cel has been FDA approved since March 2021 for RRMM after failure of ≥ 4 treatment lines containing at least one immunomodulatory agent (IMiD), a proteasome inhibitor (PI) and a CD38-targeting antibody. In February 2022 also cilta-cel received FDA approval for patients who progressed after at least 3 treatment lines including an IMiD, a PI and an anti-CD38 antibody. For both products, treating healthcare institutions must be trained and certified on the management of cytokine release syndrome (CRS) and immune effector-cell associated neurotoxicity syndrome (ICANS). In Switzerland, ide-cel is the only commercial anti-MM CAR T product available since April 2022, approved for triple-class RRMM patients who progressed after a minimum of 3 previous treatment lines.

GC012F is a dual CD19/BCMA-targeting CAR, which demonstrated promising anti-MM activity in an early phase 1 trial [11]. Multiple further anti-MM CAR T products targeting distinct combinations of MM tumor antigens, such as CD38, CD138, CD56, CS1 or integrin β7, are currently being investigated in early phase clinical trials [12]. Moreover, other novel CAR approaches for MM include allogenic CAR Ts, as well as CAR-NKs [12, 13]. In the phase 2 KarMMa (NCT03361748) study, treatment with ide-cel led to an unprecedented overall response rate (ORR) of 73% and a median overall survival of 24.8 months in a heavily pretreated patient population with relapsed/refractory multiple myeloma (RRMM) [14]. The randomized phase 3 KarMMa-3 trial (NCT03651128) recently reported improved progression-free survival (PFS) for ide-cel in RRMM compared to standard of care [15]. The efficacy of cilta-cel is being currently assessed in two first-line phase 3 trials, for patients with newly diagnosed MM, unfit or unwilling to receive treatment consolidation with high-dose chemotherapy (HDCT) and autologous stem cell transplantation (ASCT) (CARTITUDE-5, NCT04923893), and as alternative to ASCT for first line consolidation (CARTITUDE-6, NCT05257083).

In this work we report one of the first real-life cohorts of RRMM patients treated with ide-cel outside clinical trial. Moreover, since previous ide-cel studies correlated treatment efficacy with CAR T-cell expansion in vivo [14–16] and circulating soluble BCMA (sBCMA) dynamics [14–16], we analyzed these 2 parameters in our patient cohort as part of a translational co-clinical study.

## Methods

### Study design and patient cohort

Retrospective analysis of consecutive patients with triple-class exposed RRMM treated with commercial ide-cel at the University Hospital of Bern, Switzerland. Eligible patients for treatment indication had progressive disease (PD), according to the International Myeloma Working Group (IMWG) response criteria [17], following at least 3 previous treatment lines and had exposure to at least one proteasome inhibitor, one immunomodulatory agent and one anti-CD38 antibody. Further eligibility criteria were an Eastern Cooperative Oncology Group (ECOG) Performance Status of 0 or 1 at time point of treatment indication and adequate organ function, no age restrictions were defined. In patients fit to receive CAR T-cell therapy, ide-cel was prioritized over bispecific antibodies. The retrospective data collection and analysis was performed in accordance with local laws and regulations and all patients provided written informed consent.

### Response and safety assessment

MM disease responses were assessed based on bone marrow (BM) examinations and serological parameters following the IMWG standard and minimal residual disease (MRD) response criteria [17, 18]. MRD was assessed by multiparameter flow cytometry, reaching a minimal sensitivity of 1 in 10^5^ nucleated cells or higher. Toxicities were registered following the American Society for Transplantation and Cellular Therapy (ASTCT) consensus grading for CRS and immune effector-cell associated neurologic toxicities [19], and the Common Terminology Criteria for Adverse Events (CTCAE CTCAE) version 5.0 (https://ctep.cancer.gov/protocoldevelopment/electronic_applications/docs/ctcae_v5_quick_reference_5x7.pdf) for the remaining adverse events.

### Procedures

#### Lymphapheresis and anti-MM bridging therapy

Following treatment indication within a MM CAR T-cell therapy tumor board and approval of treatment reimbursement, autologous unmobilized inpatient T cell lymphapheresis was performed. To optimize T-cell quality and optimal manufacturing, a washout period of ideally 7 days for systemic treatment and therapeutic doses of systemic corticosteroids was performed. For lymphocyte collection, the continuous mononuclear cell collection procedure with the Spectra Optia (Terumo BCT) device was employed. Multi-parameter flow cytometry (BD FACSCanto™ II) was used to analyse CD3 + cell counts in peripheral blood and lymphapheresis products. After successful lymphapheresis, bridging therapy was administered as per clinical indication and following physician’s choice.

#### Treatment with ide-cel

Following delivery of the CAR T product, patients were hospitalized and lymphodepleting chemotherapy with fludarabine (30 mg/m^2^/day intravenously (iv) and cyclophosphamide (300 mg/m^2^/day iv) from day -5 to day -3 previous to ide-cel infusion (day 0) was administered. Systemic steroids were stopped 72 h prior to ide-cel administration. At day 0, 450 × 10^6^ ide-cel CAR T cells were re-infused. Following re-infusion, all patients received prophylactic pegfilgrastim on day + 1. Prophylactic anakinra 100 mg subcutaneously (sc), an interleukin-1 receptor antagonist, was administrated daily for 7 days from day 0 to + 6 based on previous studies showing lower incidence of CRS and neurotoxicity [20]. Due to risk of hypogammaglobulinemia [16], prophylactic administration of human immunoglobulins (Octagam® 30 g) was performed intravenously (iv) on day -2 and + 6. Anti-infective prophylaxis was performed with oral sulfamethoxazole-trimethoprim and valaciclovir. 14 days prior to and 6 months following ide-cel treatment, irradiated erythrocyte concentrates were administered as per clinical indication. CRS and ICANS assessment was performed every 4 h.

#### Assessment and management of CRS and ICANS

CRS and ICANS were treated following the ASCO guidelines for management of CAR T-cell therapy immune-related adverse events [21]. For CRS grading, vital parameters were assessed every 4 h following a first fever episode. For CRS grade 1, iv hydration and empiric antibiotic treatment in neutropenic patients following local guidelines was administered, as well as investigation of possible infection focus was initiated. The anti-IL6 antibody tocilizumab 8 mg/kg iv was administered in case of fever persistence over 72 h. For all patients with CRS grade 2, up-front tocilizumab 8 mg/kg iv was administered every 8 h up to a maximum of 4 doses. Dexamethasone 10 mg was administered preceding each tocilizumab dose. In patients with hypotension refractory to hydration and tocilizumab administration, the addition of vasopressors and dexamethasone 10 mg iv every 6 h was evaluated, as well as patient management within an intensive care unit (ICU). All patients with a CRS grade 3 or higher would be managed within an ICU and would receive the combination of tocilizumab and dexamethasone 10–20 mg iv every 6 h. For ICANS screening, the CARTOX-10 [22] point neurologic assessment was performed every 12 h from day 0 to + 14. In case of occurrence of new neurological symptoms, a complete neurological assessment including magnet resonance imaging, lumbar puncture and electroencephalogram was performed. ICANS management was based on administration of iv dexamethasone for all patients with ICANS grade 2–4, and selected patients with grade 1. Siltuximab (Sylvant ®), a humanized anti-IL-6 monoclonal antibody, single-dose 11 mg/kg iv (maximal dose: 1000 mg) would be administered as second line treatment for CRS and ICANS refractory to tocilizumab and dexamethasone [21]. IL-6 blood levels were monitored daily from day 0 to + 14.

### Droplet digital polymerase chain reaction (ddPCR) assay for CAR T quantification and monitoring of plasma soluble BCMA (sBCMA) levels

In previous work, we established a ddPCR assay for quantification of sequences of the intracellular domain of the bb2121 CAR-T construct. Briefly, primers and probes targeting the intracellular junction sequence between the effector (4-1BB) and co-stimulatory (CD3z) domains, were designed [23]. We performed longitudinal monitoring of circulating CAR T copies per μg of cell-free DNA (cfDNA) during up to 12 weeks following ide-cel infusion. Patients with a CAR T expansion over 10^5^ copies/μg cfDNA were defined as expanders. sBCMA plasma levels were monitored 2 to 3 times/weekly during the first week after ide-cel infusion, then every 2 week and finally every 30–60 days, and up to 120 days, as previously described [23]. For sBCMA assessment, the human BCMA/TNFRSF17 ELISA Kit (EH41RB, Thermo Fisher Scientific, Waltham, MA, USA) was used. ELISA assays were performed following the manufacturer’s instructions and mean values of triplicate measurements were plotted for each sample.

## Results

### Patient baseline characteristics

The first 16 consecutive RRMM patients treated with ide-cel at the University Hospital of Bern, Switzerland, between June and October 2022, have been included in this retrospective analysis. Median age was 69 (57 – 83) years, six (38%) patients had high-risk cytogenetic alterations, three (19%) patients had an initial R-ISS stage III and 5 (31%) extramedullary disease. Median number of previous treatment lines was 6 (3–12), including bridging therapies. Five (31%) patients had high tumor burden, defined as = / > 50% plasma cell BM infiltration, previous to ide-cel treatment. 16/16 patients (100%) had received at least one course of HDCT and ASCT. 1/16 (6%) patient additionally received a previous allogenic stem cell transplantation. No patients received previous therapy with bispecific antibodies. Patient baseline characteristics are summarized in Table 1.

**Table 1:** Patient baseline characteristics

**Characteristic**	*n* = 16
Age, median (range. yrs)	68.6 (56.7 – 82.6)
Sex, female/male (%)	5/11 (31/69)
R-ISS disease stage, n (%)	
I	6 (38)
II	7 (44)
III	3 (19)
Cytogenetik alterations, n (%)	
High risk	6 (38)
del(17p)	*4 (25)*
t(4;14)	*2 (13)*
t(14;16)	*0 (0)*
Standard risk	9 (56)
Unknown	1 (6)
Time from initial MM diagnosis to treatment with ide-cel, median (range, yrs)	7.7 (2.1–16.7)
Previous anti-myeloma regimens, median (range)	6 (3–12)
Previous ASCT, median (range)	1.5 (1–4)
Patients with Allo-SCT, n (%)	1 (6)
ECOG status, n (%)	
0	11 (69)
1	4 (25)
2	1 (6)
High tumor burden, n (%)	5 (31)
Elevated serum ferritin, n (%)	9 (56)
High D-Dimers, n (%)	9 (56)
Extramedullary disease, n (%)	5 (31)

*Abbreviations*: *Allo-SCT* Allogeneic stem cell transplantation, *ASCT* Autologous stem cell transplantation, *MM* Multiple myeloma, *R-ISS* Revised international staging system

### Production success rate and efficacy of ide-cel

**Fig. 1:**
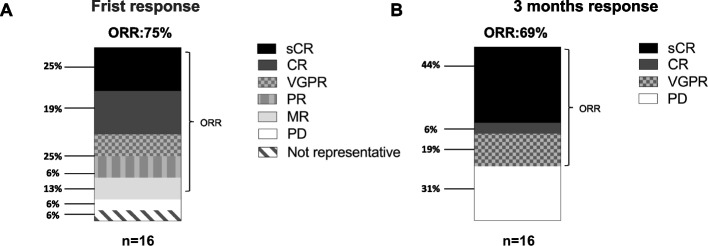
Multiple myeloma responses at 3 months follow-up. **A** First response assessment performed at a median of 12 (10–35) days after ide-cel infusion; **B** 3 months follow-up response. For response assessment, the IMWG criteria have been used, and MRD negativity was assessed by multiparameter flow cytometry. Abbreviations: CR: complete response; MR: minimal response; ORR: objective response rate; PD: progressive disease; PR: partial response; sCR: stringent complete response; VGPR: very good partial response

Overall production success rate was 88%; one (6%) patient received an out-of-specification (OOS) product, one (6%) patient required a second apheresis due to insufficient production quality. Median time from lymphapheresis to ide-cel infusion for the whole cohort was 7 (7–11) weeks. Median duration of hospitalization for ide-cel treatment was 18 (16–41) days. Preliminary response data are available from all 16 patients, assessed in a first BM aspirate performed at a median of 12 (10–35) days after ide-cel infusion and with a second BM aspirate at 10–12 weeks (3 months assessment). In one (6%) patient with prolonged hematologic toxicity, the 3 months BM assessment was performed earlier, at 9 weeks follow-up. The objective response rate (ORR) for the entire cohort was 75% in the initial assessment and 69% at 3 months assessment. At 3 months follow-up, seven (44%) patients achieved an MRD negative complete response (MRDneg CR) or stringent complete response (sCR), one (6%) patient a complete response (CR), three (19%) patients a very good partial response (VGPR), and five (31%) showed progressive disease (PD). Interestingly, two patients with CR and PR, respectively, in the first BM assessment, showed MRD negativity at the 3 months assessment (Fig. 1A, B). Despite sample size limitations, 4/6 (67%) patients with high-risk cytogenetics showed PD after treatment with ide-cel, while only 1/10 (10%) patients with standard-risk cytogenetics showed PD. No negative correlation with presence of extramedullary disease was observed. Median follow-up was 5.7 months (r: 0.6–9.0).

### Safety

**Fig. 2:**
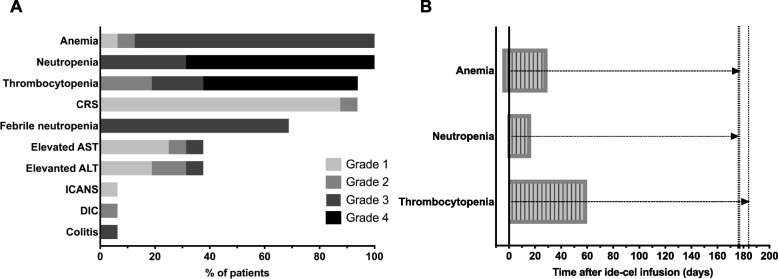
Adverse events following ide-cel administration. **A** Frequency and grade of presentation, **B** Median time to onset and duration of hematologic toxicity. Dashed lines show the upper limit of the median duration range. Abbreviations: CRS: cytokine release syndrome; DIC: disseminated intravascular coagulation; ICANS: immune effector cell-associated neurotoxicity syndrome

Observed adverse event profile was similar to previously published trial data [9]. CRS occurred in 15 patients (94%: 88% grade 1, 6% grade 2, no grade 3/4) and ICANS occurred uniquely in one patient (6% grade 2, no grade 1/3/4). Median time to CRS onset was 0 days (r: 0–2). Serum IL-6 levels correlated with CRS onset. Following CRS onset, median time to IL-6 peak-level was 3 days (r: 1–36) and median peak value 2131 pg/ml (r: 96–16,189). 15 (94%) patients required at least one dose of tocilizumab, 2 (13%) patients required the addition of dexamethasone, and 1 (6%) patient required siltuximab due to ICANS grade 2. In this patient, onset of ICANS occurred 8 days after ide-cel infusion. Hematologic toxicity was observed in all patients. Any grade of anemia occurred in 16 patients (100%: 6% grade 1, 6% grade 2, 88% grade 3), neutropenia in 16 (100%: 31% grade 3, 69% grade 4) and thrombocytopenia in 15 (94%: 19% grade 2, 19% grade 3 and 56% grade 4). Febrile neutropenia occurred in 11 (69%) patients, and infections with identification of a germ in 5 (31%) (Fig. 2A). Median onset of hematologic toxicity presented at day -5 before ide-cel administration for anemia, starting during conditioning chemotherapy, at day -1 for neutropenia and at day 0 for thrombocytopenia. Median duration of anemia was 35 days (r: 10–177), of neutropenia 18 days (r: 2–176) and of thrombocytopenia 60 days (r: 14–184) (Fig. 2B). Prolonged hematologic toxicity with anemia, neutropenia and/or thrombocytopenia of at least grade 3 persisting 3 months post-treatment with ide-cel occurred in 4/16 (25%) patients. One of the 6 patients presented a severe biphasic pancytopenia, with late-onset peak detected at day 36 post-ide-cel infusion and still ongoing at 11 weeks follow-up. BM examination in this patient showed a sCR and an aplastic BM. To date, the patient continues receiving regular transfusions, granulocyte-colonies stimulating factors, as well as supplementation with vitamin B12 and folic acid. Other common non-hematological toxicities were elevated ALT (38%: 19% grade 1, 13% grade 2, 6% grade 3, no grade 4), elevated AST (38%: 31% grade 1, 6% grade 3, no grade 2/4). One patient had a colitis (6%, grade 3) and another patient a disseminated intravascular coagulation (DIC) (6%, grade 3) (Fig. 2A). Hepatotoxicity, colitis and DIC resolved to baseline. One patient presented multi-factorial physical deterioration despite confirmed sCR in early BM biopsy and lack of infectious complications. Following patient’s wish, supportive therapy was interrupted, and the patient died 10 weeks after ide-cel treatment.

### CAR T expansion and sBCMA plasma dynamics correlate with tumor responses

**Fig. 3:**
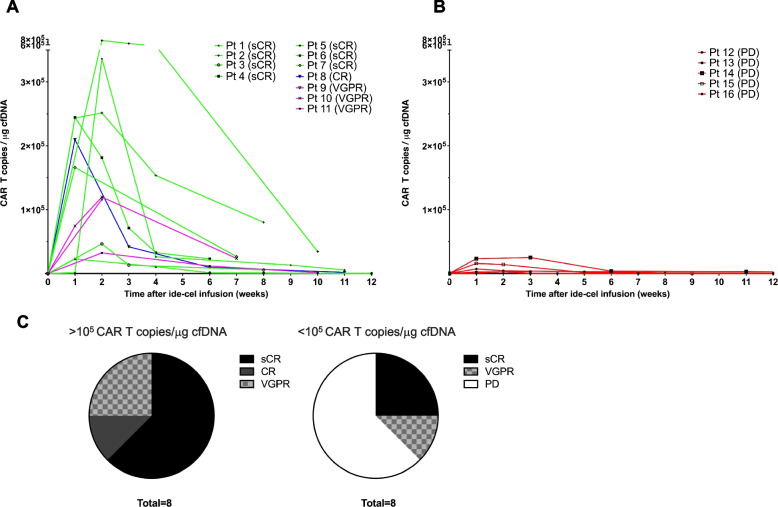
Longitudinal monitoring of circulating CAR T transgenes and correlation with tumor responses. **A** CAR T transgenes ddPCR monitoring and correlation with tumor responses in subgroup of patients who achieved an objective response following treatment with ide-cel. The observed results suggest that higher expansion levels in the first 4 weeks following ide-cel infusion correlate with better tumor responses. **B** CAR T transgenes ddPCR dynamics for the 5 patients showing PD. The observed results suggest that higher expansion levels in the first 4 weeks following ide-cel infusion correlate with better tumor responses. Green: patients with sCR; Blue: patients with CR; Magenta: patients with VGPR; red: patients with PD. **C** Tumor responses in patients with higher CAR T expansion (> 10^5^ CAR T copies/μg cfDNA) vs lower CAR T expansion (< 10^5^ CAR T copies/μg cfDNA). Abbreviations: CR: complete response; PD: progressive disease; sCR: stringent complete response; VGPR: very good partial response

**Fig. 4:**
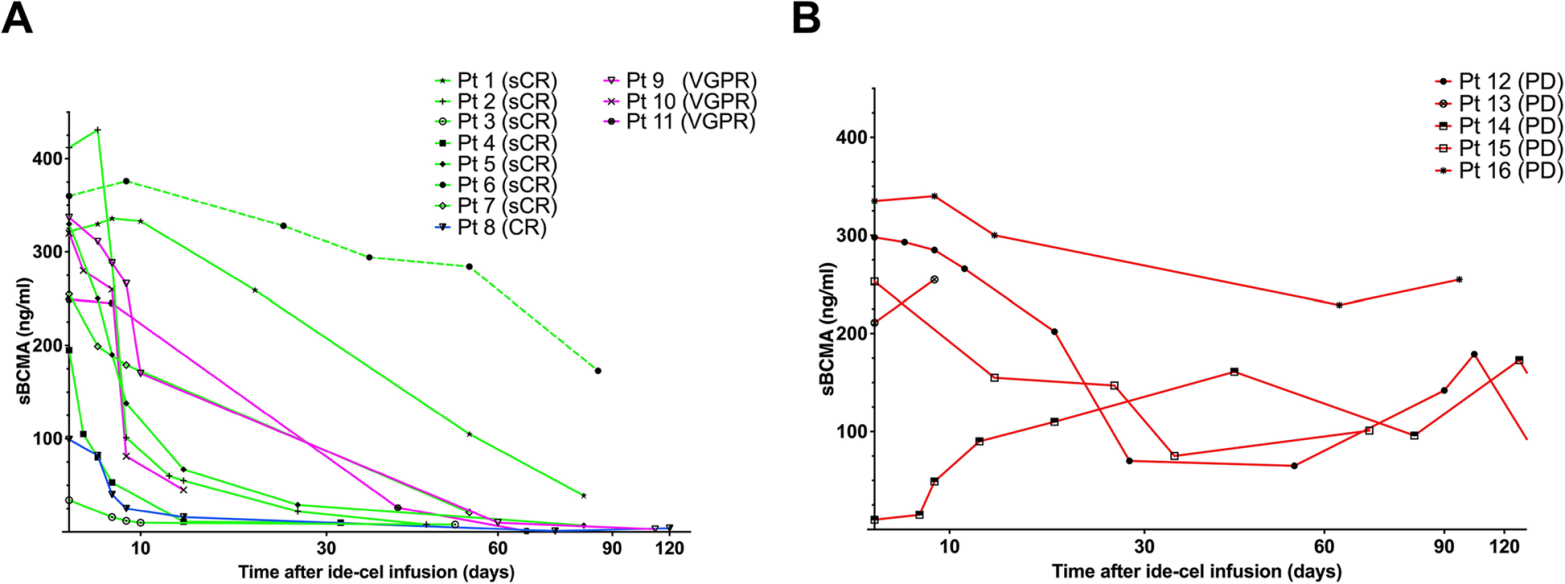
sBCMA plasma levels dynamics following ide-cel infusion. Longitudinal monitoring of sBCMA plasma levels using the human BCMA/TNFRSF17 ELISA assay and correlation with tumor responses **A** in patients reaching an objective response and **B** in non-responders. Green: patients with sCR; Blue: patients with CR; Magenta: patients with VGPR; red: patients with PD. Abbreviations: CR: complete response; PD: progressive disease; sCR: stringent complete response; VGPR: very good partial response

Longitudinal monitoring in peripheral blood of circulating CAR T copies per μg of cfDNA up to 12 weeks following ide-cel infusion is represented in Fig. 3A (responders) and 3B (non-responders). CAR T expansion peak was observed between week 1 and 3 for most patients. 8/16 patients (50%) achieved expansion over 10^5^ copies/μg cfDNA. 8/8 (100%) expanders vs 3/8 (38%) non-expanders had an objective response, 5/8 (63%) expanders vs 2/8 (25%) non-expanders had a sCR, and all patients with PD showed a CAR T expansion under 10^5^ copies/μg cfDNA (Fig. 3C). sBCMA plasma levels following ide-cel infusion are available all 16 patients, with nadir sBCMA levels observed between week + 8 and + 12 following ide-cel infusion. Non-responders showed typically lack of decrease or plateau sBCMA levels. In responders, however, high inter-patient variability regarding timing of sBCMA decrease was observed (Fig. 4A, B).

### Clinical response of an index case with extramedullary disease

**Fig. 5:**
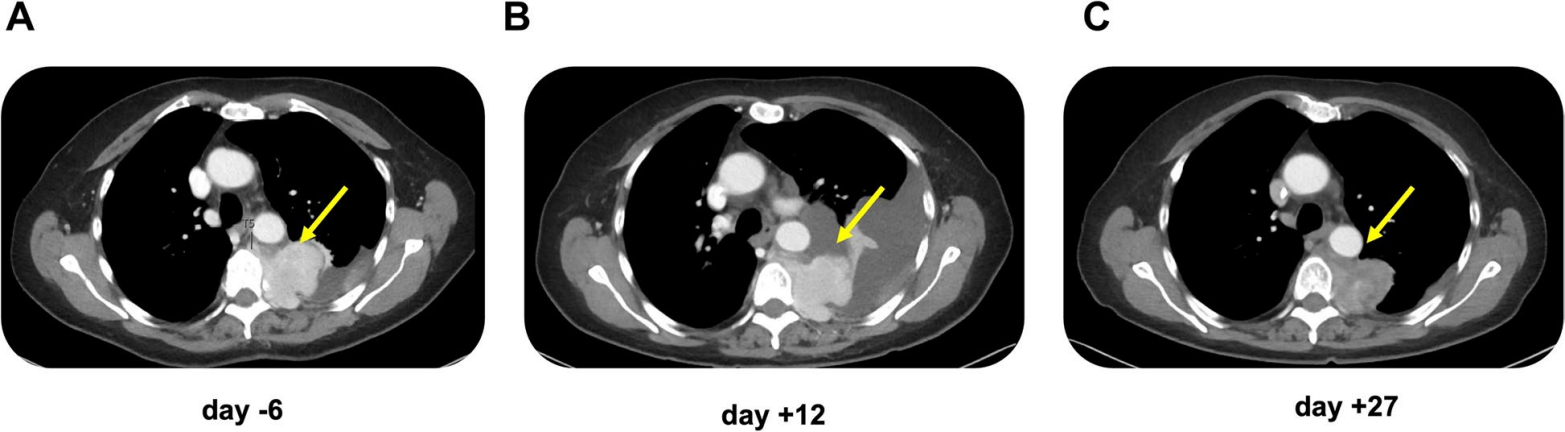
Radiographical response of extramedullary disease after ide-cel treatment in index patient with pleural extramedullary disease. CT scans (transversal) showing radiographical disease course at day -6 (**A**), + 12 (**B**) and + 27 (**C**) after ide-cel infusion. At day + 12 an initial increase in pleural infiltrative mass and pleural effusion is observed, followed by decrease of both at day + 27

A 75-year-old female patient with IgA kappa multiple myeloma, initial R-ISS Stage II, and with PD after previous 12 treatment lines, including HD-CT and ASCT. Previous to ide-cel therapy, a 12^th^ treatment line with belantamab mafodotin, carfilzomib and dexamethasone was performed, with lack of tumor response. BM biopsy before ide-cel treatment showed a 30% infiltration and a pleural biopsy confirmed presence of pleural extramedullary disease. 12 days after ide-cel infusion, patient reported progressive dyspnea, and thorax computer tomography revealed progression of the pleural infiltrative mass (70 × 48 mm, previous size 68 × 47 mm), as well as of the associated pleural effusion. Cytology and flow cytometry of pleural effusion confirmed presence of aberrant plasma cells (26.5%). 4 weeks following ide-cel infusion a slight reduction of the pleural mass could be radiographically documented (65 × 43), remaining stable 8 weeks after ide-cel (Fig. 5A, B, C). An additional radiotherapy to the pleural mass was performed. Early BM response assessment showed a PR and 3 months assessment a sCR.

## Discussion

We report one of the first real-life cohorts of RRMM patients treated with commercial ide-cel at a single Swiss academic center between June and October 2022, following the administrative approval of ide-cel in April 2022. Our patient population was comparable to the patient population of previously published trial data on ide-cel [16], with 38% of patients harboring high-risk cytogenetics, 19%, R-ISS stage III, 31%, extramedullary disease, and a median number of previous treatment lines of 6 (r: 3–12). Similarly, safety and primary response results were comparable to previous ide-cel studies [14–16].

In our patient cohort, we performed a first BM response assessment 2 weeks after ide-cel treatment (median: 12 days, r: 10–35), and further assessments at 3 and 6 months. Remarkably, 44% of patients achieved CR (including 25% of sCRs by multiparameter flow cytometry) as early as 2 weeks after ide-cel infusion. Additionally, we observed an increased depth of responses at 3 months follow-up, with 2 additional patients with initial CR and PR, respectively, further improving to an sCR. Moreover, patients showing an early sCR or CR in the first BM biopsy assessment, maintained this response status in the following response assessment 3 months after ide-cel infusion. The reported index clinical case illustrates however that an initial pseudoprogression, in this case of extramedullary lesions, might be observed in the initial weeks following CAR T-cell therapy treatment, followed by subsequent regression of these lesions. Similar experiences have been reported previously for B-cell non-Hodgkin lymphoma and B-cell lymphoblastic leukemia with extramedullary disease [24, 25].

In line with ide-cel trial data, peak CAR T expansion in the peripheral blood occurred between week + 1 and + 3 after ide-cel infusion [9]. Similar CAR T expansion dynamics have been observed for CD19-targeting CAR T-cell agents in diffuse large B-cell lymphoma (DLBCL) and B-acute lymphoblastic leukemia (B-ALL) studies [26–29]. Interestingly, in our cohort, we observed sCR and CR more frequently in patients with a CAR T expansion in the peripheral blood > 10^5^ copies/μg cfDNA as documented by ddPCR, and all patients with PD expanded < 10^5^ copies/μg cfDNA.

Additionally, we performed longitudinal monitoring of circulating sBCMA levels in peripheral blood, and found lack of progressive decrease or plateau levels in patients with PD. In contrast, responders showed constantly decreasing sBCMA levels with a nadir between week + 8 and + 12 following ide-cel infusion. This suggests that circulating sBCMA levels can be potentially used as additional biomarker to monitor MM responses following CAR T-cell therapy.

In this cohort, CAR T-associated toxicity was manageable. However, we observed expected adverse events frequently, requiring complex and timely multidisciplinary management [29–31]. Similarly to previous reports, the vast majority of patients (94%) presented CRS, mainly grade 1 (88%), and required the administration of at least one dose of tocilizumab. Thus, albeit neurotoxic adverse events presented less frequently in our cohort than previously described in patients receiving ide-cel [9], with only one (6%) patient presenting an ICANS grade 2, adequate and timely management of CAR T patients in experienced centers is essential. For instance, one patient developed refractory CRS and ICANS grade 2 requiring the administration of tocilizumab, dexamethasone and siltuximab [21]. The same patient presented the highest CAR T expansion in the peripheral blood observed in our cohort, reaching a peak of 757′927 copies /μg cfDNA 2 weeks after ide-cel infusion, which also correlated with CR and MRD negativity in the early BM assessment.

Hematologic toxicity of any grade presented in all patients, and most patients presented grade 3 or higher events. Relevantly, 25% of patients presented prolonged grade 3 or higher hematologic toxicity, persisting at 3 months follow-up after ide-cel treatment. No clear correlation with age was observed. This prolonged toxicity, especially in patients with severe pancytopenia, may represent a major clinical challenge. Prolonged hematologic toxicities persisting at > 90 days post-CAR T-cell therapy, mainly thrombocytopenia and neutropenia, have been reported in 7–38% and 0–33%, respectively, in DLBCL and B-ALL studies with tisagenlecleucel [26, 27], axicabtagene ciloleucel [32] and lisocabtagene maraleucel [33]. In the KarMMa trial, 41% of patients showed at least grade 3 persistent neutropenia, and 49% thrombocytopenia, with a median time to recovery of 1.9 and 2.1 months, respectively [9, 34]. Moreover, in the KarMMa trial 3/127 (0.2%) of patients required stem cell support due to prolonged pancytopenia [9]. The physiopathology of this post CAR-T persistent cytopenia is incompletely understood, and the clinical management relies on supportive measures, mainly transfusions, use of hematopoietic growth factors and hematopoietic stem cell boost [35].

Regarding the ide-cel manufacturing process, the median time from lymphapheresis to ide-cel infusion was 7 (7–11) weeks. This seemed acceptable, since most patients (68.8%) were able to receive a bridging therapy, while patients with lower tumor burden did not require bridging. Manufacturing success rate was 88%, which is clearly lower as compared to previously published data [17]. In the KarMMa phase 2 trial, only one case of production failure out of 140 included patients was reported (99.3% production success rate) [17]. In this trial, the number of previous treatment lines, as well as the proportion of patients with a history of HDCT with ASCT, was comparable to our cohort. Thus, we hypothesized that the lower real-life manufacturing success rate was not related to a more heavily pretreated patient population. For tisagenlecleucel, the latest reported manufacturing success rates were around 96%, with less than 3% of patients receiving OOS products [36]. Further reports from real-life cohorts could clarify the maximal expected production success rate for MM CAR-Ts and the possible underlying factors.

## Conclusions

In summary, results from this real-life cohort suggest that treatment with ide-cel for RRMM outside of a clinical trial is feasible and leads to an ORR of 69% in a heavily pretreated patient population. The safety profile was manageable; however, prolonged hematologic toxicity remains a major clinical challenge. Tumor responses were more frequent in patients with higher CAR T-cell expansion by ddPCR assessment in the peripheral blood, and circulating sBCMA levels correlated with BM responses. Future studies should further investigate strategies to enhance CAR T expansion in vivo in order to optimize and maintain treatment efficacy.

## Data Availability

The datasets used and/or analysed during the current study are available from the corresponding author on reasonable request.

## Abbreviations

ASCT: Autologous stem cell transplantation
ASTCT: American Society for Transplantation and Cellular Therapy
B-ALL: B-acute lymphoblastic leukemia
BCMA: B-cell maturation antigen
BM: Bone marrow
cfDNA: Cell-free DNA
CAR: Chimeric antigen receptor
cilta-cel: Ciltacatagene-autocel
CR: Complete response
CRS: Cytokine release syndrome
CTCAE: Common Terminology Criteria for Adverse Events
ddPCR: Droplet digital polymerase chain reaction
DLBCL: Diffuse large B-cell lymphoma
ECOG: Eastern Cooperative Oncology Group
HDCT: High-dose chemotherapy
ide-cel: Idecabtagenum vicleucelum
ICANS: Immune effector-cell associated neurotoxicity syndrome
ICU: Intensive care unit
IMWG: International Myeloma Working Group
iv: Intravenously
MM: Multiple myeloma
MRD: Minimal residual disease
MRDneg CR: MRD negative complete response
ORR: Overall response rate
PD: Progressive disease
PFS: Progression-free survival
R-ISS: Revised Multiple Myeloma International Staging System
RRMM: Relapsed/refractory multiple myeloma
sBCMA: Soluble BCMA
sc: Subcutaneously
sCR: Stringent complete response (sCR)
VGPR: Very good partial response

## References

[1] Sterner RC, Sterner RM (2021). CAR-T cell therapy: current limitations and potential strategies. Blood Cancer J.

[2] Larson RC, Maus MV (2021). Recent advances and discoveries in the mechanisms and functions of CAR T cells. Nat Rev Cancer.

[3] Muhammad N, Mao Q, Xia H (2017). CAR T-cells for cancer therapy. Biotechnol Genet Eng Rev.

[4] Gill S, Brudno JN (2021). CAR T-Cell Therapy in Hematologic Malignancies: Clinical Role, Toxicity, and Unanswered Questions. Am Soc Clin Oncol Educ Book.

[5] Gagelmann N, Riecken K, Wolschke C, Berger C, Ayuk FA, Fehse B (2020). Development of CAR-T cell therapies for multiple myeloma. Leukemia.

[6] Nydegger A, Novak U, Kronig MN, Legros M, Zeerleder S, Banz Y (2021). Transformed lymphoma is associated with a favorable response to CAR-T-Cell treatment in DLBCL patients. Cancers (Basel).

[7] Anagnostou T, Brody JD (2022). In CAR T cell-treated lymphomas, the T cell rich get richer. Nat Med.

[8] Shah N, Chari A, Scott E, Mezzi K, Usmani SZ (2020). B-cell maturation antigen (BCMA) in multiple myeloma: rationale for targeting and current therapeutic approaches. Leukemia.

[9] Munshi NC, Anderson LD, Shah N, Madduri D, Berdeja J, Lonial S (2021). Idecabtagene Vicleucel in Relapsed and Refractory Multiple Myeloma. N Engl J Med.

[10] Brechbühl S, Bacher U, Jeker B, Pabst T (2021). Real-World Outcome in the pre-CAR-T Era of Myeloma Patients Qualifying for CAR-T Cell Therapy. Mediterr J Hematol Infect Dis.

[11] Du J, Jiang H, Dong B, Gao L, Liu L, Ge J (2022). Updated results of a multicenter first-in-human study of BCMA/CD19 dual-targeting fast CAR-T GC012F for patients with relapsed/refractory multiple myeloma (RRMM). J Clin Oncol.

[12] Teoh PJ, Chng WJ (2021). CAR T-cell therapy in multiple myeloma: more room for improvement. Blood Cancer J.

[13] Shah UA, Mailankody S (2020). CAR T and CAR NK cells in multiple myeloma: expanding the targets. Best Pract Res Clin Haematol.

[14] Usmani S, Patel K, Hari P, Berdeja J, Alsina M, Vij R (2022). KarMMa-2 cohort 2a: efficacy and safety of idecabtagene vicleucel in clinical high-risk multiple myeloma patients with early relapse after frontline autologous stem cell transplantation. Blood.

[15] Rodriguez-Otero P, Ailawadhi S, Arnulf B, Patel K, Cavo M, Nooka AK (2023). Ide-cel or standard regimens in relapsed and refractory multiple myeloma. N Engl J Med.

[16] Munshi NC, Larry D.Anderson J, Shah N, Jagannath S, Berdeja JG, Lonial S,  (2020). Idecabtagene vicleucel (ide-cel; bb2121), a BCMA-targeted CAR T-cell therapy, in patients with relapsed and refractory multiple myeloma (RRMM): Initial KarMMa results. J Clin Oncol.

[17] Kumar S, Paiva B, Anderson KC, Durie B, Landgren O, Moreau P (2016). International myeloma working group consensus criteria for response and minimal residual disease assessment in multiple myeloma. Lancet Oncol.

[18] Durie BG, Harousseau JL, Miguel JS, Bladé J, Barlogie B, Anderson K (2006). International uniform response criteria for multiple myeloma. Leukemia.

[19] Lee DW, Santomasso BD, Locke FL, Ghobadi A, Turtle CJ, Brudno JN (2019). ASTCT consensus grading for cytokine release syndrome and neurologic toxicity associated with immune effector cells. Biol Blood Marrow Transplant.

[20] Park JH, Sauter CS, Palomba ML, Shah GL, Dahi PB, Lin RJ (2021). A phase II study of prophylactic anakinra to prevent CRS and neurotoxicity in patients receiving CD19 CAR T cell therapy for relapsed or refractory lymphoma. Blood.

[21] Santomasso BD, Nastoupil LJ, Adkins S, Lacchetti C, Schneider BJ, Anadkat M (2021). Management of immune-related adverse events in patients treated with chimeric antigen receptor T-Cell therapy: ASCO guideline. J Clin Oncol.

[22] Neelapu SS, Tummala S, Kebriaei P, Wierda W, Gutierrez C, Locke FL (2018). Chimeric antigen receptor T-cell therapy — assessment and management of toxicities. Nat Rev Clin Oncol.

[23] Seipel K, Porret N, Wiedemann G, Jeker B, Bacher VU, Pabst T (2022). sBCMA plasma level dynamics and Anti-BCMA CAR-T-Cell treatment in relapsed multiple myeloma. Curr Issues Mol Biol.

[24] Danylesko I, Shouval R, Shem-Tov N, Yerushalmi R, Jacoby E, Besser MJ (2021). Immune imitation of tumor progression after anti-CD19 chimeric antigen receptor T cells treatment in aggressive B-cell lymphoma. Bone Marrow Transplant.

[25] Huang J, Rong L, Wang E, Fang Y (2021). Pseudoprogression of extramedullary disease in relapsed acute lymphoblastic leukemia after CAR T-cell therapy. Immunotherapy.

[26] Schuster SJ, Bishop MR, Tam CS, Waller EK, Borchmann P, McGuirk JP (2018). Tisagenlecleucel in adult relapsed or refractory diffuse large B-Cell lymphoma. N Engl J Med.

[27] Maude SL, Laetsch TW, Buechner J, Rives S, Boyer M, Bittencourt H (2018). Tisagenlecleucel in children and young adults with B-Cell lymphoblastic leukemia. N Engl J Med.

[28] Locke FL, Miklos DB, Jacobson CA, Perales M-A, Kersten M-J, Oluwole OO (2021). Axicabtagene ciloleucel as second-line therapy for large B-Cell lymphoma. N Engl J Med.

[29] Pabst T, Joncourt R, Shumilov E, Heini A, Wiedemann G, Legros M (2020). Analysis of IL-6 serum levels and CAR T cell-specific digital PCR in the context of cytokine release syndrome. Exp Hematol.

[30] Messmer AS, Que YA, Schankin C, Banz Y, Bacher U, Novak U (2021). CAR T-cell therapy and critical care : a survival guide for medical emergency teams. Wien Klin Wochenschr.

[31] Gössi S, Bacher U, Haslebacher C, Nagler M, Suter F, Staehelin C (2022). Humoral responses to repetitive doses of COVID-19 mRNA vaccines in patients with CAR-T-Cell therapy. Cancers (Basel).

[32] Neelapu SS, Locke FL, Bartlett NL, Lekakis LJ, Miklos DB, Jacobson CA (2017). Axicabtagene ciloleucel CAR T-Cell therapy in refractory large B-Cell lymphoma. N Engl J Med.

[33] Abramson JS, Palomba ML, Gordon LI, Lunning MA, Wang M, Arnason J (2020). Lisocabtagene maraleucel for patients with relapsed or refractory large B-cell lymphomas (TRANSCEND NHL 001): a multicentre seamless design study. The Lancet.

[34] Logue JM, Peres LC, Hashmi H, Colin-Leitzinger CM, Shrewsbury AM, Hosoya H (2022). Early cytopenias and infections after standard of care idecabtagene vicleucel in relapsed or refractory multiple myeloma. Blood Adv.

[35] Corona M, Shouval R, Alarcón A, Flynn J, Devlin S, Batlevi C (2022). Management of prolonged cytopenia following CAR T-cell therapy. Bone Marrow Transplant.

[36] Rodrigues M, Duran E, Eschgfaeller B, Kuzan D, Habucky K (2021). Optimizing commercial manufacturing of tisagenlecleucel for patients in the US: a 4-year experiential journey. Blood.

